# Combinatorial stress responses: direct coupling of two major stress responses in *Escherichia coli*

**DOI:** 10.15698/mic2014.09.168

**Published:** 2014-09-01

**Authors:** Daniel R. Brown, Geraint Barton, Zhensheng Pan, Martin Buck, Sivaramesh Wigneshweraraj

**Affiliations:** 1 MRC Centre for Molecular Bacteriology and Infection, Imperial College London, SW7 2AZ, UK.; 2 Centre for Systems Biology and Bioinformatics, Division of Biosciences, Imperial College London, SW7 2AZ, UK.; 3 Department of Life Sciences, Imperial College London, SW7 2AZ, UK.

**Keywords:** stringent response, NtrC, transcription, sigma 54, RNA polymerase, (p)ppGpp

## Abstract

Nitrogen is an essential element for all life, and this is no different for the bacterial cell. Numerous cellular macromolecules contain nitrogen, including proteins, nucleic acids and cell wall components. In *Escherichia coli *and related bacteria, the nitrogen stress (Ntr) response allows cells to rapidly sense and adapt to nitrogen limitation by scavenging for alternative nitrogen sources through the transcriptional activation of transport systems and catabolic and biosynthetic operons by the global transcriptional regulator NtrC. Nitrogen-starved bacterial cells also synthesize the (p)ppGpp effector molecules of a second global bacterial stress response - the stringent response. Recently, we showed that the transcription of *relA*, the gene which encodes the major (p)ppGpp synthetase in *E. coli*, is activated by NtrC during nitrogen starvation. Our results revealed that in *E. coli* and related bacteria, NtrC functions in combinatorial stress and serves to couple two major stress responses, the Ntr response and stringent response.

The synthesis of (p)ppGpp in response to nutritional stresses has been studied for more than forty years. The regulatory basis by which (p)ppGpp levels are modulated by the (p)ppGpp synthetase RelA (product of *relA* gene) and the (p)ppGpp synthetase/hydrolase SpoT (product of *spoT* gene) in *E. coli* are well understood: upon amino acid starvation, the binding of uncharged (deacylated) tRNAs in the ‘A’ site of the ribosome leads to the stalling of the protein synthesis machinery and thereby compromises translation. RelA detects and interacts with the stalled ribosomes, and this stimulates its enzymatic activity to synthesize (p)ppGpp. Accumulation of (p)ppGpp has far-reaching consequences on major cellular processes, including transcription, translation and DNA replication, which collectively form the cell's stringent response. The stringent response leads to a down-regulation of stable RNA synthesis (rRNA & tRNA), required in high abundance for fast-growing cells, whilst biosynthetic operons are up-regulated to promote survival until growth conditions improve. The transcriptional inhibition of stable RNA synthesis has a knock-on effect of releasing the RNA polymerase (RNAp) from these highly transcribed genes and thereby allowing adaptive reprogramming of gene expression to cope with the stress. Importantly, the RelA-mediated stringent response is at the heart of bacterial adaptation to starvation and stress, and plays a major role in the bacterial cell cycle and expression of virulence genes in bacterial pathogens.

One objective of our study was to explore evidence for links between the perception of nitrogen stress and expression of *relA* in *E. coli*. Since (p)ppGpp levels are known to increase in response to nitrogen limitation, we wanted to elucidate how amino acid starvation that occurred as a result of nitrogen starvation might lead to changes in the transcription of *relA*. The Ntr response in *E. coli* is regulated by the two component system NtrBC, where NtrB is the sensor histidine kinase. Via the PII protein NtrB senses the nitrogen status of the cell and responds to low combined nitrogen availability by phosphorylating, and thereby activating, the DNA-binding transcription factor NtrC. As a bacterial enhancer binding protein (bEBP), NtrC specifically activates transcription in an ATP consuming reaction from bacterial promoters that are bound by RNAp containing the major variant promoter-specificity sigma (σ) factor 54 (σ^54^). In response to nitrogen starvation, NtrC reprograms transcription by directly activating approximately 45 genes, which allows the expression of scavenging and transport systems for alternative nitrogen sources, genes that encode proteins involved in catabolism of nitrogenous compounds and the glutamine biosynthesis pathway to enhance growth in low nitrogen environments.

To investigate the link between Ntr stress response and *relA* transcription at the genome-wide level, we used chromatin immunoprecipitation followed by high-throughput sequencing (ChIP-seq) to determine the genome-wide binding of NtrC under conditions of nitrogen availability and nitrogen starvation. Simultaneously, we also determined the genome-wide binding of the RNAp, which was used as a proxy to report gene expression i.e. transcription. The majority of the genomic loci bound by NtrC under nitrogen starvation were upstream of promoters of genes that were previously demonstrated or predicted to be activated by NtrC. Strikingly, the results also identified one new binding site for NtrC, which was located upstream of *relA*. We next conducted a series of experiments to elaborate this finding: (1) Using recombinant and *in situ* activated NtrC we were able to demonstrate *in vitro* that NtrC can specifically bind to a DNA fragment representing the region upstream of *relA*. (2) *In vivo* gene expression data revealed a >2-fold increase in *relA* mRNA levels upon transition of *E. coli* cells growing under nitrogen replete conditions to nitrogen starved conditions. Concurrently, and as expected, a >2-fold increase in the intracellular levels of RelA protein was detected. These differences were not detected in the absence of *glnG*, the gene encoding NtrC, but were restored to wild-type levels upon complementation with plasmid-borne *glnG*. (3) We identified a novel σ^54^-depedent promoter and showed *in vitro* and *in vivo* that it drives the transcription of *relA* in a strictly NtrC and σ^54 ^dependent manner.

During stringent response (p)ppGpp reprograms transcription by binding to the RNAp and thereby affecting occupancy and usage of so called ‘stringent’ promoters to modulate gene expression. Therefore, we determined RNAp occupancy and activity at all known stringent promoters in nitrogen starved *E. coli* cells to gauge if NtrC activated *relA* leads to the stringent response. Results revealed the expected RNAp occupancy and activity at ~60% of stringent promoters, whereas ~35% of stringent promoters showed no change whilst the remaining 5% showed an opposite effect to that expected. Thus, it is possible that (p)ppGpp only modulates activity of a subset of stringent promoters in nitrogen starved *E. coli*. Nevertheless, the results unequivocally demonstrated that in nitrogen starved *E. coli* cells, NtrC and σ^54^ control the increased transcription of *relA* for the accumulation of (p)ppGpp (Figure 1). Intriguingly, results also revealed that the transcription of 9 of the 12 toxin-antitoxin operons, which are indirectly upregulated by ppGpp in *E. coli*, were activated under nitrogen starvation. As TA’s play a central role in the acquisition of bacterial persistence, we propose that NtrC-mediated accumulation of (p)ppGpp could lead to the "activation" or "priming" of persistence traits in response to nitrogen starvation.

**Figure 1:**
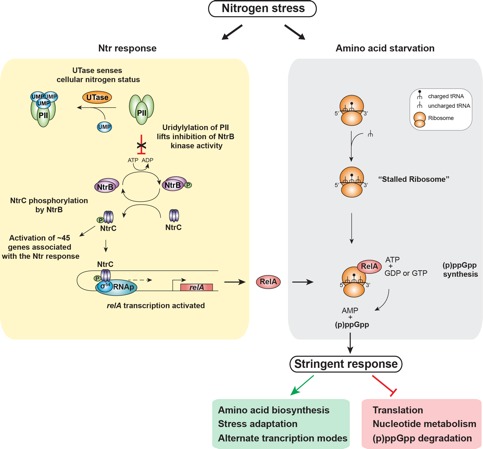
FIGURE 1: NtrC-activated gene expression of *relA* links the Ntr and stringent responses during nitrogen stress. See main text for details.

Previous studies from the Kustu lab dubbed the Ntr stress response as a scavenging response as many of the genes that are activated by NtrC encode transport systems for nitrogenous compounds. Our new results have highlighted the need for *E. coli *to integrate the requirement of scavenging for new nitrogen sources with stringent-response-mediated changes in gene expression to allow the bacterial cell to cope with low nitrogen availability (Figure 1). The clear increase in NtrC mediated activation of *relA* transcription in nitrogen starved *E. coli *is likely to significantly increase intracellular levels of (p)ppGpp. Whether (p)ppGpp, in addition to mediating the stringent response, also binds to proteins specifically associated with the Ntr response and modulates their activity post-transcriptionally remains to be determined. However, this is a very likely possibility, given that (p)ppGpp has been documented to bind to GdhA, an enzyme involved in nitrogen metabolism, in order to signal the ClpAP protease to degrade GdhA during nitrogen starvation. Further, our results have also underscored the fundamental importance of σ^54^ in bacterial stress response. Given that there are at least ten bEBPs in *E. coli*, which rely on σ^54^ and activate the transcription of genes that allow the cells to cope with a variety of nutritional and abiotic stresses, an attractive line of future investigation would be to explore interactions between these adaptive responses and the stringent response via mechanisms such as that evident with the Ntr response.

